# Intraocular lens power calculations in eyes with pseudoexfoliation syndrome

**DOI:** 10.1038/s41598-021-98675-5

**Published:** 2021-09-24

**Authors:** Aleksandra Wlaź, Agnieszka Kustra, Agnieszka Rozegnał-Madej, Tomasz Żarnowski

**Affiliations:** Department of Diagnostics and Microsurgery of Glaucoma, Medical University, Chmielna 1, 20-079 Lublin, Poland

**Keywords:** Eye diseases, Lens diseases

## Abstract

To compare refractive outcomes after cataract surgery in pseudoexfoliation syndrome (PEX) and control eyes and to investigate the accuracy of 3 intraocular lens (IOL) calculation formulas in these eyes. In this prospective comparative study 42 eyes (PEX group) and 38 eyes (control group) of 80 patients were included. The follow-up was 3 months. The refractive prediction error (RPE), mean absolute error (MAE), median absolute error (MedAE) and the percentages of eyes within ± 0.25 D, ± 0.5 D, ± 1.0 D and ± 2.0 D of prediction error were calculated. Three IOL calculation formulas (SRK/T, Barrett Universal II and Hill-RBF) were evaluated. PEX produced statistically significantly higher mean absolute errors and lower percentages of eyes within ± 0.5 D than control eyes in all investigated IOL calculation formulas. There were no statistically significant differences in the median absolute errors between the 3 formulas in either PEX or control eyes. Refractive outcomes after cataract surgery are statistically significantly worse in PEX than in control eyes. All three IOL calculation formulas produced similar results in both PEX and control eyes.

**Trial registration:** ClinicalTrials.gov registration number NCT04783909.

## Introduction

With modern phacoemulsification surgical techniques, cataract surgery might be considered the most common refractive procedure^1^. Patients have increasingly higher refractive expectations, and to meet them, we require a greater level of accuracy in postoperative refractive outcomes. Evolution of optical biometry and intraocular lens (IOL) power calculation formulas over the recent years have led to decrease in rate of refractive surprises. Nevertheless, accurate prediction of effective lens position (ELP) still remains a biggest source of error. There are many models for the prediction of postoperative anterior chamber depth (ACD) in pseudophakic eye, including corneal curvature and corneal height, preoperative ACD and lens thickness (LT). Therefore, ELP is formula-dependent and does not reflect true postoperative ACD. Most of modern IOL power calculation formulas are based on theoretical eye model and are vergence formulas based on Gaussian optics^2,3^. These are Sanders, Retzlaff, Kraff/theoretical (SRK/T)^4^ and Barrett Universal II^5^. In contrast, the Hill-Radial Basis Function (Hill-RBF)^6^ is not theoretical vergence formula but is based on artificial intelligence and regression analysis of a very large database of postoperative refractive outcomes.

Precise estimation of ELP may be extremely difficult in cases with zonular dehiscence and capsular weakness. Pseudoexfoliation (PEX) syndrome, first characterized by Lindberg in 1917^7^, is considered a risk factor in cataract surgery because it may pose intraoperative and postoperative challenges. There are many intraoperative complications including zonular dialysis, capsule tear/rupture, vitreous loss, and dropped nucleus^8–12^ that are more common in PEX eyes. However, improvements in techniques and phacoemulsification in experienced hands may result in low incidence of complications. Nevertheless, zonular dehiscence and capsular weakness may have large contribution to ELP and refractive outcome of the surgery.

The purpose of this study was to evaluate the refractive outcomes of cataract surgery in PEX syndrome and determine which of the commonly used IOL formulas (SRK/T, Barrett Universal II and Hill-RBF) is the best in predicting postoperative refractive outcomes in PEX.

## Patients and methods

This was a prospective comparative study of 80 eyes of 80 patients with senile cataract who underwent uncomplicated sutureless phacoemulsification surgery with lens implantation in the Department of Diagnostics and Microsurgery of Glaucoma of Medical University of Lublin, Poland between October 2016 and December 2017. The protocol of this study was approved by the Bioethics Committee of Medical University in Lublin (Poland) and registered under the number KE-0254/241/2014. Written informed consent was obtained from all the participants in accordance with tenets of Declaration of Helsinki.

Group 1 comprised 42 eyes with PEX syndrome and coexisting cataract and group 2 comprised 38 eyes with cataract only. The follow up was 3 months.

### Inclusion and exclusion criteria

The inclusion criteria of the study were patients aged over 50 years with visually disabling cataract and pseudoexfoliation (PEX) without glaucoma. Cataract grading was performed using LOCS III (Lens Opacities Classification System III) scale^13^.

Exclusion criteria included corneal pathology, glaucoma, corneal astigmatism greater than 2.0 diopters (D), previous eye surgery or subjects with decreased vision due to other reasons than cataract (e.g., exudative age-related macular degeneration (AMD), proliferative diabetic retinopathy, inflammatory eye diseases), intraoperative complications, postoperative corrected distance visual acuity (CDVA) worse than 20/40, axial length below 21 mm and above 25 mm, dense cataracts or poor fixation requiring ultrasound biometry. We also excluded eyes with manifest iridophacodonesis and those in which a capsular tension ring was inserted. Our selection criteria for the study subjects and methods followed the recommendations of protocols regarding best practices for studies of IOL formulas^14–17^.

### Surgical procedure

All surgeries were performed using Infiniti (Alcon laboratories, Inc., Fort Worth, TX, USA) under topical anaesthesia by one surgeon (TŻ) through 2.2 mm incision in the upper corneal limbus. The hydroimplantation of Aspira-aA (HumanOptics AG, Erlangen, Germany) to the capsular bag was performed in all cases.

### Intraocular lens power calculation

Ocular biometry was performed in all eyes using PCI (Version V.7.7 IOL Master 500, Carl Zeiss Meditec AG, Jena, Germany). The IOL power for implantation was selected using power calculations from SRK/T. Keratometry, ACD and axial length (AL) data were exported from PCI to Excel spreadsheet (version 16.44 for Mac, Microsoft Corp.) and formatted for statistical analysis. Three IOL power calculation formulas were evaluated: SRK/T, Barrett Universal II and Hill-RBF. A-constant for SRK/T and Hill-RBF was 118.7. The lens factor was 1.73 for the Barrett Universal II. The PCI software was used for calculations of SRK/T formula. Data were manually entered into the online Barrett Universal II^18^ and Hill-RBF calculator^6^ by one investigator (AK) and results were checked for plausibility by another investigator (AW).

### Postoperative refraction

Manifest refraction was measured postoperatively at 3 months by the same investigator. The testing distance was 6 meters (m), as suggested by Simpson and Charman^19^.

### Main outcome measures

The refractive prediction error (RPE) was calculated as the difference between the measured and predicted postoperative refractive spherical equivalent. A negative RPE shows a more myopic result, and a positive RPE represents a more hyperopic shift. To eliminate the systematic error, the mean RPE for each formula was zeroed out by adjusting the RPE for each eye. After this adjustment, the mean absolute error (MAE) and median absolute error (MedAE) were calculated. The MAE was defined as the mean absolute value of the adjusted RPE and MedAE was defined as the median absolute value of the adjusted RPE. Outcomes before the RPE adjustment are also presented because there is controversy about optimization to be used in atypical eyes and it is suggested that both kinds of results should be reported^16^. The percentages of eyes within ± 0.25 D, ± 0.5 D, ± 1.0 D and ± 2.0 D of prediction error were calculated.

The primary outcome measure was to compare refractive outcomes (MAE, MedAE, percentage of eyes within certain range of prediction error) in PEX and control eyes. The secondary outcome measure was to determine whether any of IOL power prediction formulas (SRK/T, Barrett Universal II and Hill-RBF) is more precise for these challenging eyes.

### Power and sample size calculation

To detect a difference of one standard deviation of RPE, assuming a standard deviation (SD) of 0.3 D, a two-sided α error of 5% and achieving a power of 80%, the sample size of 70 eyes (35 eyes in each group) was calculated.

### Statistical analysis

The statistical analysis was performed using SPSS for Mac (version 26.0.0.1; IBM SPSS Statistics, Chicago, IL, USA). Differences were considered statistically significant at *p* < 0.05. Normality of data was assessed using Kolmogorov–Smirnov test. For continuous variables between the study groups, the Mann–Whitney U-test was used for analysis. Categorical variables were analysed using the Fisher-exact test. To compare the accuracies of the three formulas, Friedman nonparametric test of the MedAE was used. To compare the percentage of eyes within a certain range of prediction errors between the three formulas, Cochran Q test was performed. The Bonferroni correction was applied for multiple comparisons. For comparison of the preoperative ACD and RPE, univariate linear regression analysis and Pearson’s correlation analysis were performed.

## Results

42 eyes (PEX group) and 38 eyes (control group) of 80 patients were enrolled to the study. All patients completed the follow-up schedule of 3 months and were analysed. No posterior capsule opacification (PCO), IOL tilt or decentration were observed during the follow-up.

The patients’ demographics are shown in Table 1. No statistically significant differences between the groups in the age, sex, right/left eye, mean axial length, mean corneal power and mean IOL power were noted (p > 0.05). The mean ACD was smaller in PEX group, and this difference was statistically significant (*p* = 0.012).

**Table 1:** Baseline characteristics.

Characteristic	Mean ± SD		*p* value
	PEX *n* = 42	Control *n* = 38	
Age (y)	77.26 ± 6.97	74.21 ± 9.90	0.198^a^
Sex (male/female)	15/27	20/18	0.176^b^
Right/left eye	21/21	23/15	0.376^b^
AxL (mm)	23.38 ± 0.75	23.48 ± 1.01	0.784^a^
ACD (mm)	2.98 ± 0.48	3.19 ± 0.36	0.012^a^
K (D)	43.95 ± 1.65	43.56 ± 1.53	0.525^a^
IOL power (D)	21.13 ± 1.61	21.42 ± 2.53	0.242^a^
LOCS score (NS)	2.64 ± 0.73	2.60 ± 0.72	0.811^a^

AxL: axial length; ACD: anterior chamber depth; K: keratometry; IOL: intraocular lens; y: year; mm: millimeter; D: diopter; LOCS: Lens Opacities Classification System III; NS: nuclear sclerosis.

^a^Mann–Whitney U test; ^b^Fisher’s exact test.

Due to an incorrect formula constant, systematic bias in refractive prediction were found in all formulas and mean RPEs from all three formulas were statistically above zero thus giving hyperopic results (*p* < 0.05 in all formulas in PEX and control eyes, Wilcoxon signed rank test). The MedAE without adjusting the RPE to zero are shown in Table 2. There were statistically significant differences between PEX and control groups in all three formulas (SRK/T *p* = 0.014, Barrett Universal II *p* = 0.004, Hill-RBF *p* = 0.001; Mann–Whitney U test). No statistically significant differences were found between formulas in PEX eyes (*p* = 0.570, Friedman test), however in control eyes SRK/T had a statistically significantly higher MedAE than the Barrett Universal II and Hill-RBF (0.001, Friedman test). Post-hoc analysis with Wilcoxon signed-rank test was conducted with a Bonferroni correction applied, resulting in a significance level set at p < 0.017. There was no significant difference between Barrett Universal II and Hill-RBF (Z = − 0.735, *p* = 0.462). There were statistically significant differences between SRK/T and Barrett Universal II (Z = − 2.543, *p* = 0.011) and between SRK/T and Hill-RBF (Z = − 2.981, *p* = 0.003).

**Table 2:** Refractive prediction error, mean absolute error and median absolute error produced by each formula in PEX and control groups without adjusting mean RPE to zero.

	SRK/T	Barrett Universal II	Hill-RBF	*P* value (Friedman test)
**PEX *n* = 42**				
RPE ± SD, D (range)	0.321 ± 0.644 (1.440, + 1.710)	0.322 ± 0.641 (− 1.490, + 1.970)	0.283 ± 0.643 (− 1.500, + 1.970)	
MAE ± SD, D	0.589 ± 0.406	0.569 ± 0.431,	0.566 ± 0.420	
MedAE, D	0.505	0.505	0.410	0.570
**Control *n* = 38**				
RPE ± SD, D (range)	0.303 ± 0.381 (− 0.480, + 1.355)	0.203 ± 0.371 (− 0.580, + 1.355)	0.188 ± 0.379 (− 0.570, + 1.225)	
MAE ± SD, D	0.379 ± 0.303	0.320 ± 0.273	0.312 ± 0.283	
MedAE, D	0.333	0.315	0.185	0.001

PEX: pseudoexfoliation syndrome; RPE: refractive prediction error; SD: standard deviation; D: diopter; MAE: mean absolute error; MedAE: median absolute error.

After adjusting the mean RPE to zero for all three formulas, which would eliminate the systematic error from incorrect formula constant, Table 3 presents the mean refractive prediction error (RPE), SD and range of prediction error, MedAE and MAE determined by 3 formulas in PEX and control groups. In both PEX and control groups, there were no significant differences in MedAE between the three formulas (*p* = 0.636 and *p* = 0.442, respectively, Friedman test). However, in PEX group the lowest MedAE was found in Barrett Universal II formula, without statistical significance. There were statistically significant differences between PEX and control groups in all three formulas (SRK/T *p* = 0.010, Barrett Universal II *p* = 0.042, Hill-RBF *p* = 0.039; Mann–Whitney U test).

**Table 3:** Refractive prediction error, mean absolute error and median absolute error produced by each formula in PEX and control groups after adjusting mean RPE to zero.

	SRK/T	Barrett Universal II	Hill-RBF	*P* value (Friedman test)
**PEX *n* = 42**				
RPE ± SD, D (range)	0.000 ± 0.644 (− 1.761, + 1.389)	0.000 ± 0.641 (− 1.812, + 1.648)	0.000 ± 0.643 (− 1.783, + 1.687)	
MAE ± SD, D	0.498 ± 0.401	0.479 ± 0.420,	0.479 ± 0.420	
MedAE, D	0.460	0.385	0.400	0.636
**Control n = 38**				
RPE ± SD, D (range)	0.000 ± 0.381 (− 0.783, + 1.052)	0.000 ± 0.371 (− 0.783, + 1.152)	0.000 ± 0.379 (− 0.758, + 1.038)	
MAE ± SD, D	0.288 ± 0.244	0.286 ± 0.232	0.292 ± 0.237	
MedAE, D	0.213	0.219	0.243	0.442

PEX: pseudoexfoliation syndrome; RPE: refractive prediction error; SD: standard deviation; D: diopter; MAE: mean absolute error; MedAE: median absolute error.

**Figure 1:**
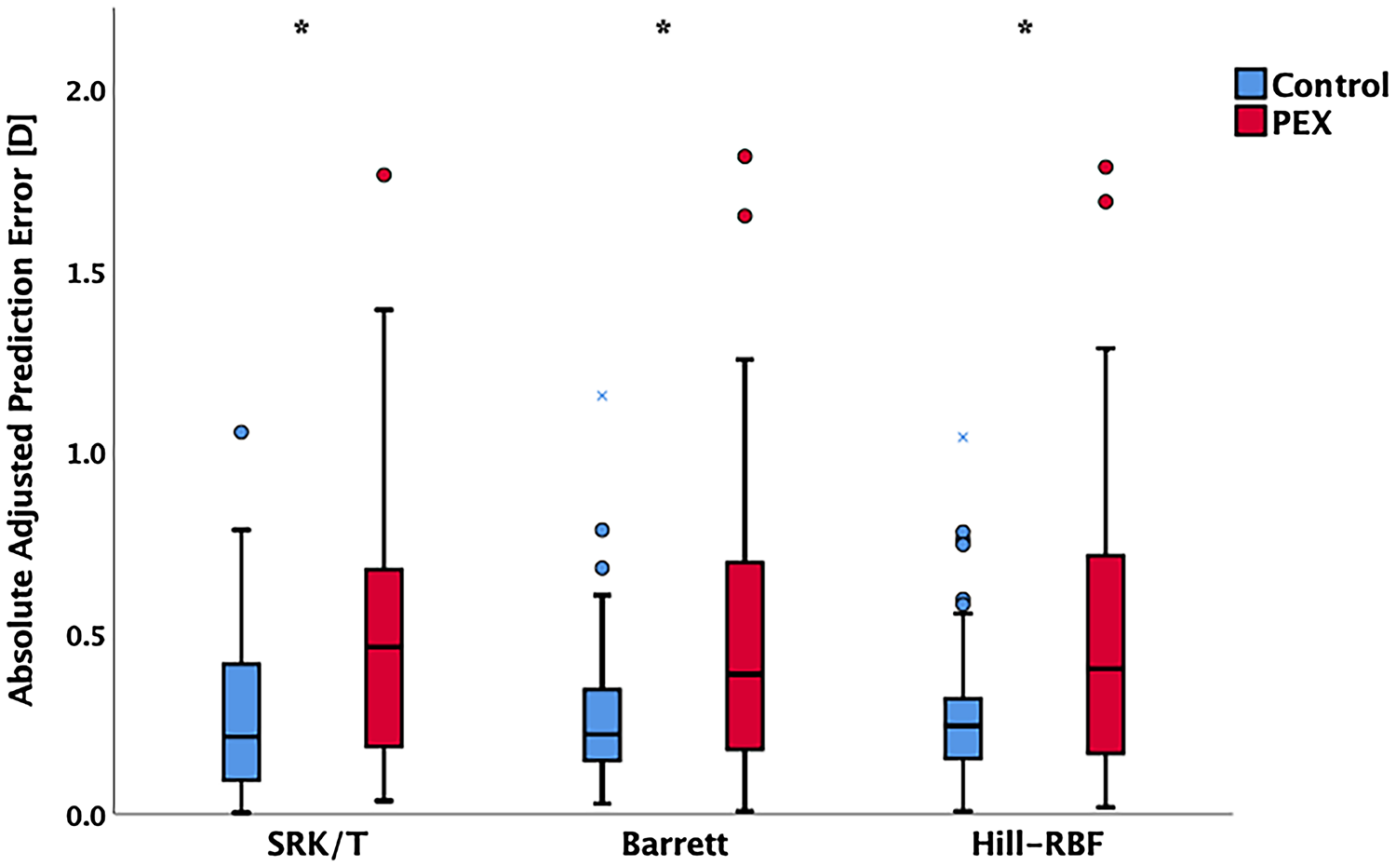
Box plot presentation of the absolute error (after adjusting the mean numerical refractive prediction error to zero) of IOL calculation formulas in PEX and control groups. There were statistically significant differences between PEX and control groups in all three formulas. Median values (horizontal lines), 25/75 percentiles (boxes), 5/95 percentiles (whiskers) and outliers (dots). *p < 0.05 PEX versus Control group (Mann–Whitney U test); IOL = intraocular lens; PEX = pseudoexfoliation syndrome.

All three formulas produced higher absolute errors in PEX groups than in control group (Fig. 1).

**Figure 2:**
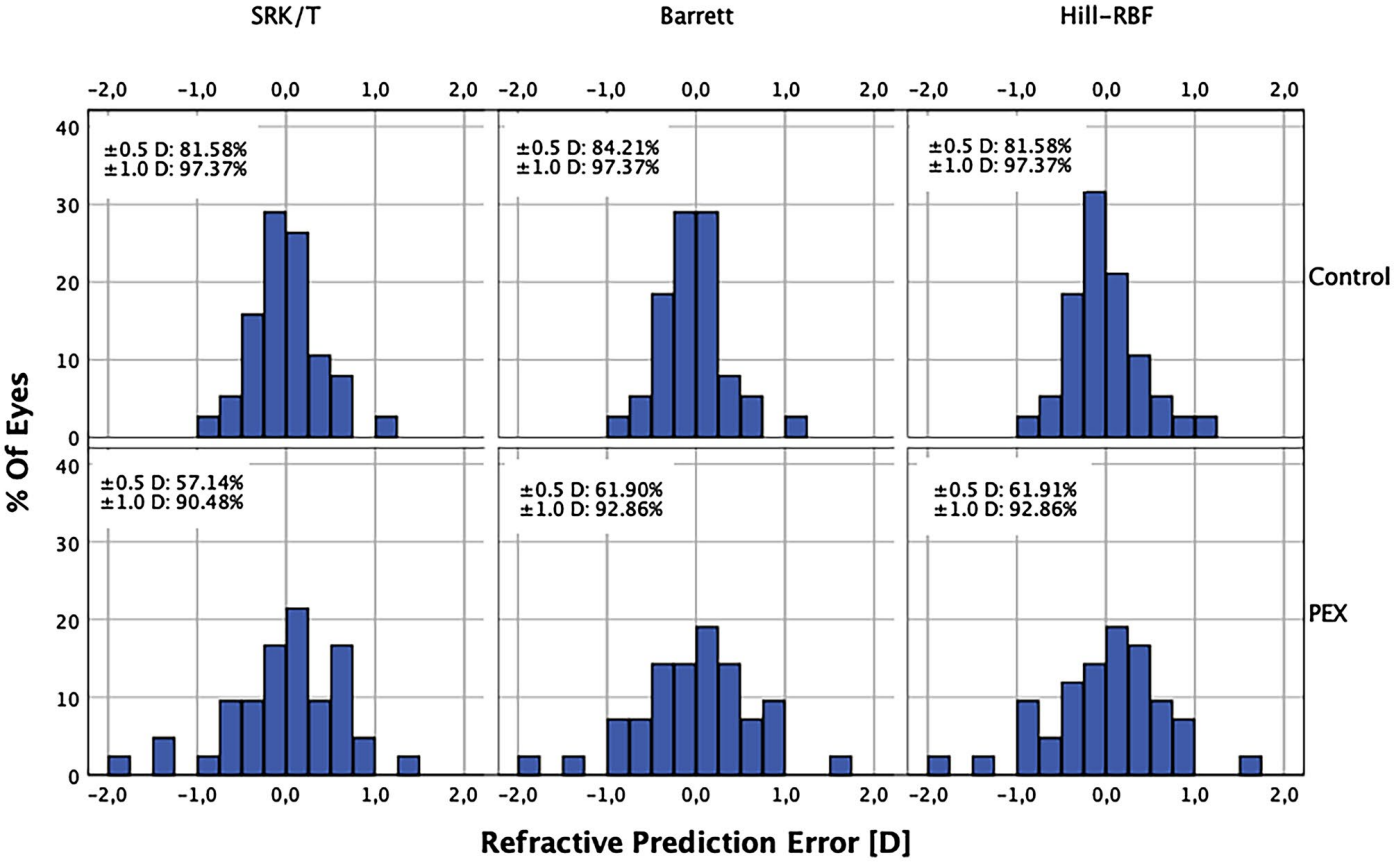
Histogram comparing the percentage of cases within a given diopter range of refractive prediction error for three IOL power calculation formulas in PEX and control group. No significant differences were found among the three formulas using Cochran Q test (all *p* > 0.05) in either PEX or control eyes. IOL = intraocular lens; PEX = pseudoexfoliation syndrome.

The percentage of eyes within a certain range of prediction errors is shown in Table 4 and Fig. 2. The percentages of eyes within ± 0.25 D, ± 0.5 D, ± 1.0 D and ± 2.0 D were not significantly different among the three formulas using Cochran Q test (all *p* > 0.05) in both PEX and control groups. In PEX group percentages of eyes within ± 0.5 D were significantly smaller than in control group using all three formulas (Fisher’s exact test).

**Table 4:** Percentage of eyes with refractive prediction errors within ± 0.25 D, ± 0.5 D, ± 1.0 D and ± 2.0 D after adjusting the mean numerical refractive prediction errors to zero (Fisher’s exact test).

Prediction error within	SRK/T			Barrett Universal II			Hill-RBF		
	Control *n* (%)	PEX *n* (%)	p	Control *n* (%)	PEX *n* (%)	p	Control *n* (%)	PEX *n* (%)	p
± 0.25 D	21 (55.26)	16 (38.10)	0.178	22 (57.89)	14 (33.33)	0.042*	20 (52.63)	14 (33.33)	0.113
± 0.5 D	31 (81.58)	24 (57.14)	0.029*	32 (84.21)	26 (61.90)	0.044*	31 (81.58)	26 (61.91)	0.044*
± 1.0 D	37 (97.37)	38 (90.48)	0.362	37 (97.37)	39 (92.86)	0.617	37 (97.37)	39 (92.86)	0.617
± 2.0 D	38 (100.00)	42 (100.00)	–	38 (100.00)	42 (100.00)	–	38 (100.00)	42 (100.0)	–

PEX: pseudoexfoliation syndrome; D: diopter; * p < 0.05.

**Figure 3:**
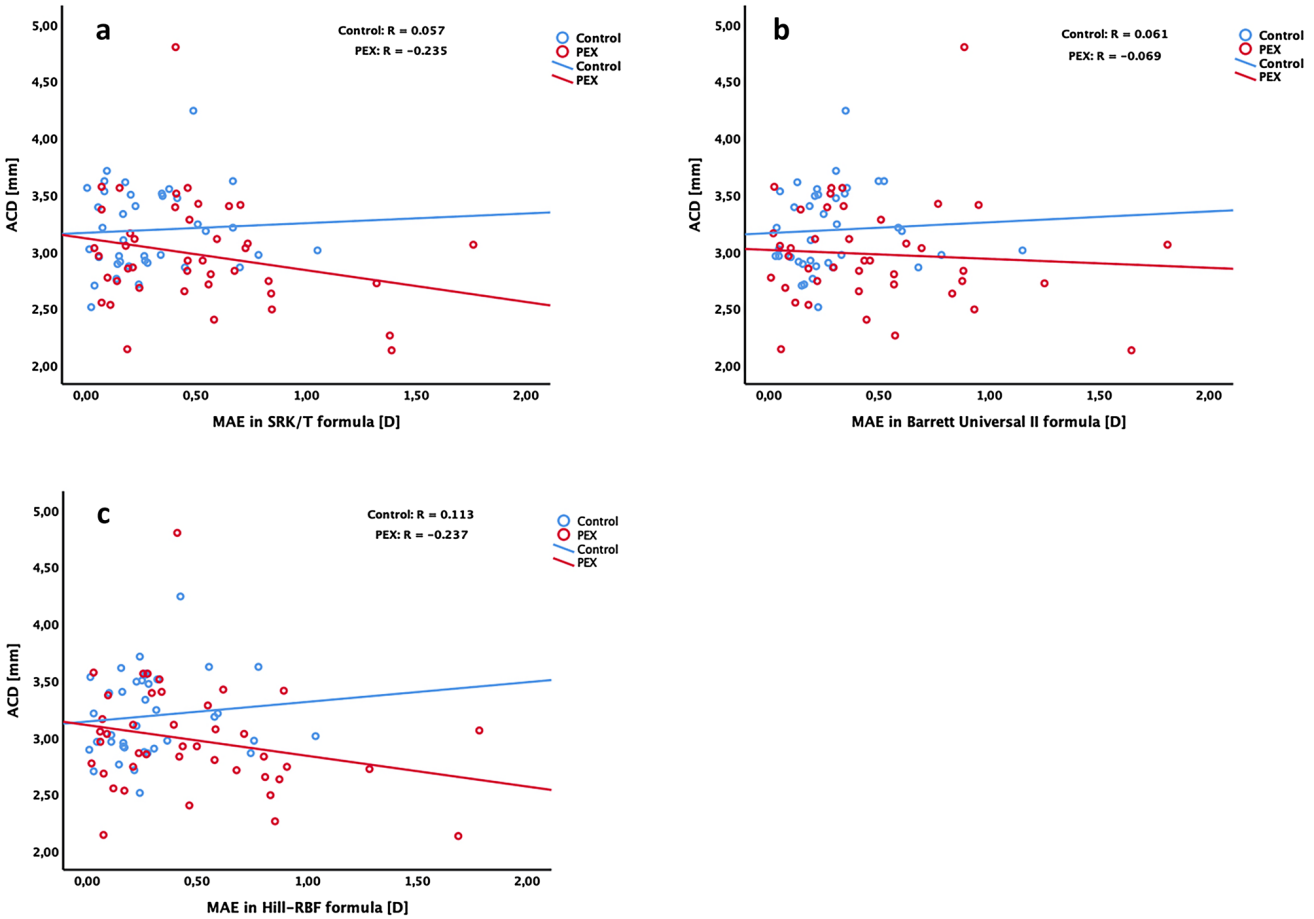
Scatterplots showing correlations between preoperative ACD and MAE in SRK/T (**a**), Barrett Universal II (**b**) and Hill-RBF (**c**) formulas. No significant association between preoperative ACD and MAE was found in any of evaluated IOL calculation formulas in either PEX or control group. Pearson’s correlation analysis revealed trend of negative correlation between preoperative ACD and MAE in SRK/T and Hill-RBF formulas in PEX eyes.

The results of linear regression analysis revealed no significant association between preoperative ACD and MAE in any of evaluated IOL calculation formulas in either PEX or control group (Fig. 3). Despite lack of statistical significance, Pearson’s correlation analysis revealed trend of negative correlation between preoperative ACD and MAE in SRK/T (R = − 0.235, *p* = 0.067) and Hill-RBF formulas (R = − 0.237, *p* = 0.065) in PEX eyes. No correlation was observed between preoperative ACD and MAE in Barrett Universal II formula in PEX eyes (R = − 0.069, *p* = 0.332). In control eyes no correlation was found in any of evaluated IOL calculation formulas (SRK/T: R = 0.057, *p* = 0.367; Barrett Universal II: R = 0.061, *p* = 0.358, Hill-RBF: R = 0.113, *p* = 0.250).

## Discussion

It is well known that in PEX the risk of zonular instability is high and it may lead to refractive surprises after cataract surgery. Our study provides insight into refractive outcomes after cataract surgery in PEX syndrome. Our results show that patients with PEX have more unpredictable refractive outcomes than patients without PEX.

The refractive outcome after phacoemulsification cataract surgery is affected by many factors. Currently, it is believed that the stability of postoperative IOL position represented by effective lens position (ELP) and postoperative ACD is one of the key factors in determining refractive outcome of the surgery^20,21^. Olsen^21^ found that 42% of IOL power prediction errors were caused by incorrect estimation of postoperative ACD and 1.0 mm error in estimated postoperative ACD is equivalent to an error of about 1.5 D in the spectacle plane.

With current technology, the accuracy of IOL calculation formulas is limited by their approach to predict the ELP. Precise prediction of ELP is more difficult in eyes with PEX where zonular dehiscence and capsular weakness is more common than in healthy eyes. The aim of our study was also to determine whether new IOL power prediction formulas might improve outcomes in these challenging eyes. According to Holladay et al.^22^ SD of RPE is the single most accurate measure when comparing IOL power calculation formulas. Although we found higher SDs of RPE in PEX than in control group, we found no differences among three formulas in PEX and control eyes separately. We thought that new IOL calculation formulas may perform better in PEX eyes. Especially the Hill-RBF formula which is an alternative to the existing theoretical vergence formulas. In this method there is no estimation of ELP, as in traditional formulas, which we thought might be beneficial in eyes with zonular instability and difficulties with estimating postoperative ACD. However, we found no significant differences between formulas. The possible reason for this may be small number of eyes and further studies on larger samples may be required.

To eliminate the systematic error from an incorrect formula constant, we adjusted the individual RPE by the amount equal to mean RPE in order to produce mean RPE of zero. This solution was proposed by Hoffer et al.^14,15^ and is required before evaluating refractive outcomes. After optimizing the lens constant so that the mean RPE is equal to zero, the SD is more affected with outliers than MedAE, since SD is related to the square value of the difference of each value of the mean. The MedAE represents more central location of the absolute errors, but in our opinion, in non-typical eyes, it is essential to also compare SDs as they represent outliers because these are outcomes we want to avoid in IOL power calculations.

It has been reported that in eyes with PEX syndrome, preoperative ACD is significantly smaller than in healthy eyes. The possible explanation for this finding may lay in ciliary zonular laxity, more common in PEX patients. In a study by Doganay et al.^23^, ACD in PEX glaucoma eyes was found to be significantly lower than in control group. Gharagozloo et al.^24^ found that anterior chamber volumes were significantly smaller in both affected and unaffected eyes with unilateral PEX compared with control eyes. In a study by Gur Gungor et al.^25^ mean preoperative ACD values in PEX group (3.04 ± 0.5 mm) were lower than the normal group (3.26 ± 0.3 mm) but the difference was not statistically significant. These findings are consistent with our results. In our study mean preoperative ACD was significantly lower in PEX than in controls.

Despite lack of statistical significance, we found trend of negative correlation between preoperative ACD and MAE in SRK/T and Hill-RBF formulas in PEX eyes. Interestingly, in Barrett Universal II there was no association between ACD and MAE. Thus, shallower ACDs may be more prone to have bigger MAE in PEX than in control eyes. Barrett Universal II may be a good choice to calculate IOL in these eyes. Further studies on larger samples are needed to draw definite conclusions.

Whereas many studies have investigated changes in ACD after phacoemulsification cataract surgery^26–29^, so far there are only two studies evaluating these changes in PEX eyes. Gur Gungor et al.^25^ compared ACD changes with Allegro Oculyzer-Scheimpflug imaging system after phacoemulsification cataract surgery in PEX and control eyes. They reported more significant ACD change in patients with PEX (0.46 ± 0.3 mm) compared with control patients (0.12 ± 0.1 mm). They also have measured postoperative refractive errors. The MAEs calculated by the SRK/T, Haigis, Hoffer and Holladay 1 formulas were higher in PEX eyes, but the difference was not statistically significant. However, the main aim of this study was to compare the ACD changes in PEX and control eyes and methods of assessment of postoperative refraction were not clearly clarified. Also, relatively small sample size (22 eyes with PEX and 30 control eyes) may be the reason why MAE did not differ significantly between the groups. Fallah Tafti et al.^30^ evaluated the pseudophakic ACD or ELP change after cataract surgery in patients with PEX using amplitude scan (A-scan) ultrasound (Echoscan) and optical coherence tomography (OCT) Visante. They found a significant increase in postoperative ACD from 1 month (3.97 ± 0.39 mm) to 6 months after cataract surgery (4.06 ± 0.36 mm). This backward movement of the IOL may be associated with concurrent refractive errors.

Ishikawa et al.^31^ compared deviations in refraction between PEX patients and controls and found no difference between groups at 1 month. However, refractive outcomes were assessed 1 month postoperatively whereas postoperative manifest refraction should be ideally measured at 3 months or later^14^, especially since the ACD may increase significantly in PEX between 1 and 6 months postoperatively^30^. Moreover, regarding the fact that more than 1 eye from one patient were included into the analysis, advanced statistical models should have been used. The SE refraction errors were distributed more widely in both myopic and hyperopic directions in PEX group, without statistical significance. However, it is not stated if the mean arithmetic RPE was equal to zero before calculations or lens constant optimization had been carried out.

Manoharan et al.^32^ have found that PEX glaucoma patients had an odds ratio (OR) of 7.3 of having a refractive surprise after phacoemulsification surgery compared with controls. In their retrospective study evaluating refractive outcomes after cataract surgery in glaucoma patients, 3 out of 11 eyes with PEX (27.3%) had refractive surprise of above 1.0 D. Higher risk of error in refractive outcome was only in chronic angle closure glaucoma (ACG), where 3 out of 7 eyes (42.9%) had refractive surprise of above 1.0 D.

Strengths of the current study are the use of homogenous group of eyes and the use of a single IOL model implanted by a single surgeon. The study was conducted with the guidelines proposed by Hoffer et al.^14,15^ in editorial and recent update on protocols for IOL power formula studies. Our study complies with the proposed protocol by using 1 eye from each study subject, adjusting the RPE to zero out the arithmetic mean error and using recommended statistical methods for analyzing these data. Data was displayed in a histogram as recommended by Reinstein et al.^33^. To the best of our knowledge, our study is the first one to report the outcomes of new IOL power calculation formulas (Barrett Universal II and Hill-RBF) in eyes with PEX.

The primary limitation of our study is the small sample size. The refractive outcomes were measured at 3 months postoperatively, whereas in PEX stabilization of refraction may last longer. The Hill-RBF method was evaluated using PCI biometry, however it was optimized for OLCR (optical low-coherence reflectometry). The biometry was performed using IOL Master 500, therefore lens thickness (LT) was not measured.

In conclusion, in this prospective comparative study, we demonstrated statistically significant differences in refractive outcomes after phacoemulsification cataract surgery between PEX and healthy eyes. We found no differences between SRK/T, Barrett Universal II and Hill-RBF IOL power calculation formulas in predicting refractive outcomes. In the current era of phacoemulsification and higher expectations from patients to achieve optimal refractive outcomes, our study shows that patients with PEX have significantly worse refractive results and further studies with larger sample size are needed to improve predicting the ELP and therefore increasing the degree of accuracy of IOL power calculation in eyes with PEX.

## Data Availability

Data are available on reasonable request from Aleksandra Wlaź (aleksandra.wlaz@icloud.com).
